# Long-term effects of vitamin D supplementation and maintaining sufficient vitamin D on knee osteoarthritis over 5 years

**DOI:** 10.1186/s13075-023-03167-8

**Published:** 2023-09-23

**Authors:** Zhiqiang Wang, Zhaohua Zhu, Feng Pan, Shuang Zheng, Venkat Parameswaran, Leigh Blizzard, Changhai Ding, Benny Antony

**Affiliations:** 1 Clinical Research Centre, Zhujiang Hospital, Southern Medical University, Guangzhou, China; 2 Menzies Institute for Medical Research, University of Tasmania, Hobart, Australia; 3 Department of Rheumatology and Immunology, Arthritis Research Institute, the First Affiliated Hospital of Anhui Medical University, Hefei, China; 4 Department of Endocrinology, Royal Hobart Hospital, Hobart, TAS 7000 Australia; 5 Department of Epidemiology and Preventive Medicine, Monash University, Melbourne, Australia

**Keywords:** Vitamin D, Osteoarthritis, Knee symptoms, Depression, Knee surgery

## Abstract

**Objectives:**

This study aimed to investigate the long-term effect of vitamin D supplementation compared to placebo over 5 years in participants with knee osteoarthritis (OA). We also aimed to describe the effect of maintaining sufficient serum vitamin D levels over five years in knee OA.

**Methods:**

Participants (*n* = 173) from the Hobart centre of the Vitamin D Effects on Osteoarthritis (VIDEO) trial were extensively followed up 3 years after the cessation of 2-year investigational treatment. Participants were classified as maintaining sufficient vitamin D (*n* = 79) and not maintaining sufficient vitamin D (*n* = 61) groups.

**Results:**

There was no significant difference in change in the knee symptoms, depression, and serum levels of IL6 and hs-CRP between both comparisons after 3 years of cessation of the clinical trial. However, among participants who reported no knee surgery (KS), there was a significant improvement in WOMAC function (*β*: − 83.7, 95% CI: − 167.3, 0) and depression scores (*β*: − 1.3, 95% CI: − 2.3, − 0.2) in vitamin D group compared to the placebo group. Similarly, those who maintained adequate vitamin D levels over 5 years had significantly less WOMAC knee pain (*β*: − 33.9, 95% CI: − 65.7, − 2) and physical dysfunction (*β*: − 105.5, 95% CI: − 198.2, − 12.8) than participants with vitamin D deficiency over 5 years.

**Conclusion:**

Vitamin D supplementation over 2 years or maintaining vitamin D sufficiency for 5 years was not associated with statistically significant differences in change in knee symptom scores over 5 years. However, among participants who did not report KS, 2-year vitamin D supplementation and maintaining sufficient vitamin D was linked to modest improvements in knee symptoms and depression scores in knee OA.

**Supplementary Information:**

The online version contains supplementary material available at 10.1186/s13075-023-03167-8.

## Highlights

• Serum vitamin D levels regressed to the mean value of around 61.4 nmol/L after 3 years of the cessation of the VIDEO trial. However, this was significantly higher than the mean baseline vitamin D levels (42.7 nmol/L) before starting the trial. Vitamin D levels decreased in the original vitamin D supplementation group and slightly increased in the placebo group over 5 years.

• Among knee osteoarthritis participants without knee surgery, vitamin D supplementation was linked to modest improvement in knee symptoms over 5 years.

• Among participants without knee surgery, maintaining sufficient vitamin D was associated with a reduction in knee symptoms over 5 years.

**Supplementary Information:**

The online version contains supplementary material available at 10.1186/s13075-023-03167-8.

## Introduction

Osteoarthritis (OA) is the most common joint disorder in adults around the world. Being a debilitating disease characterised by joint pain and physical dysfunction, OA induces some long-term consequences and comorbidities, including depression, impaired sleep, reduced work participation, and increased mortality [1, 2]. Current guidelines recommend education, structured exercise, and weight loss as core management strategies [3, 4]. There are only limited pharmacological options for the management of OA, and non-steroidal anti-inflammatory drugs (NSAIDs), commonly used by OA patients, are often associated with adverse events and suboptimal treatment effects.

Vitamin D plays an essential role in calcium homeostasis and bone metabolism [5]. After cutaneous synthesis or diet intake, vitamin D is converted to 25-hydroxy-vitamin D (25(OH)D) and subsequently to the active form 1,25(OH)_2_D [6]. By acting on vitamin D receptors, vitamin D has various biological activities in cartilage, bone, and muscle and may protect the degeneration of joint tissues in OA [7, 8]. Vitamin D deficiency [commonly defined as a serum level of 25(OH)D < 50 nmol/l] is prevalent in older people. In Australia, up to 80% of women and 70% of men living in hostels or nursing homes were deficient in vitamin D [9]. Epidemiological studies have suggested moderate evidence of association between vitamin D deficiency and progression of OA [10, 11]. Our systematic review reported a moderate evidence of the association between low vitamin D levels and progression of radiographic knee OA [12], including knee joint space narrowing (JSN) [13].

Randomised clinical trials (RCT) of vitamin D supplementation effects on symptoms and structural damage in knee OA have yielded mixed results [14], with some reporting null results [15, 16], while one study reporting a modest but statistically significant benefits [17]. Vitamin D Effects on Osteoarthritis (VIDEO) RCT from our group suggested that vitamin D treatment did not result in significant changes in MRI-measured tibial cartilage volume or knee pain over 2 years in participants with symptomatic knee OA and low serum 25-hydroxyvitamin D levels [18]. One possible reason for the lack of efficacy in our RCT may be that the VIDEO trial participants were only followed up for 2 years and the vitamin D treatment effect may have a prolonged effect [11]. Besides, an elevated vitamin D level in the placebo group (62% of participants in the VIDEO trial achieved vitamin D sufficiency over 2 years) could have diluted the differences in effects between vitamin D and placebo groups [11]. The post hoc analysis from the VIDEO trial reported beneficial effects of maintaining vitamin D sufficiency over 2 years on lower tibial cartilage volume loss, lower increase in effusion-synovitis volume, and improvement in the Western Ontario and McMaster Universities Osteoarthritis Index (WOMAC) assessed physical function [19].

Most of the vitamin D trials in knee OA indicated a consistent improvement in the knee pain score beyond the 12-month mark, as demonstrated by the data presented in their figures [11]. Conversely, the placebo group in these trials did not show any change in their knee pain score beyond 12 months [18]. Additionally, the alteration in X-ray-detected joint space narrowing, measured after 12 months, remained steady in the vitamin D group but deteriorated in the placebo group in the Oxford VIDEO trial [15]. It is worth noting that none of these studies reported a significant interaction between the treatment time and the treatment effect. While it is not possible to definitively ascertain the outcomes that might have emerged from a longer treatment duration, the potential for vitamin D to exhibit effectiveness at a later stage should not be dismissed [11].

Vitamin D supplementation may exert long-term health benefits [8]. One study indicates that buildup of reserves of vitamin D, presumably in body fat, may explain serum 25(OH)D continues to rise over several months while vitamin D3 plateaus [20]. This means vitamin D reserves in the body can help maintain adequate vitamin D levels even with temporary decreases in intake/synthesis. Vitamin D supplementation also has prolonged effects, including but not limited to, reducing the risk of falls and fractures after vitamin D supplementation from the ViDA trial [21], lowering central blood pressure parameters among vitamin D deficient participants [22], and decreasing the risk of overall mortality [23]. However, evidence for long-term effects of vitamin D supplementation on OA symptoms is scarce. Therefore, we hypothesise that the VIDEO trial participants may have a prolonged beneficial effect from vitamin D supplementation for more than 2 years.

Regardless of how adequate vitamin D levels were achieved (through supplementation or other factors), combining both vitamin D and placebo groups shows the impact of vitamin D status itself, as serum 25(OH)D is considered the ideal biomarker of vitamin D status [24]. RCTs of vitamin D routinely analyse results pooling treatment arms based on achieved 25(OH)D levels [25]. An ancillary study of the Vitamin D and Omega-3 Trial (VITAL) also analysed placebo group based on different 25(OH)D levels [26]. Combining groups also account for the natural fluctuation in levels of vitamin D over time. Vitamin D levels have substantial within-person fluctuations over time due to multiple factors [27]. Therefore, additional analyses based on the level maintained over 5 years rather than a single measurement better captures vitamin D status in our study.

Substantial heterogeneity of treatment effects was reported in patients with and without knee surgery (KS), including total knee replacement (TKR) and arthroscopy [28]. TKR followed by nonsurgical treatment resulted greater pain relief than nonsurgical treatment alone [29]. Certain post-surgical interventions, including physical therapy and rehabilitation, could also impact study outcomes and are not standardised across patients in an observational follow-up of RCTs. Surgical treatment also confounds and complicates interpretation of treatment effects [30]. Besides, there are innate differences between patients opting for surgical vs nonsurgical treatment for OA. TKR as a treatment for knee OA often represents “joint death” or “overhaul”; therefore, it is important to conduct a sub-group analysis by removing participants who underwent surgical interventions.

Overall, we aimed to investigate the effect of vitamin D supplementation compared to placebo over 2 years on knee symptoms, psychological health, and inflammatory markers over 5 years in participants with knee OA. We also aimed to describe the effect of maintaining sufficient serum vitamin D levels over 5 years on knee OA. Besides, we aimed to describe the long-term effect of vitamin D supplementation based on the status of knee surgical intervention during the follow-up period.

## Methods

### Study design and participants

This study was a 5-year extensive follow-up of the VIDEO trial [18]. The VIDEO trial was a multicentre, randomised, double-blind, placebo-controlled clinical trial conducted in Hobart (Tasmania) and Melbourne (Victoria). Four hundred thirteen participants were recruited from Hobart (*n* = 261) and Melbourne (*n* = 152). Participants with knee OA were randomised (stratified by site) to the vitamin D supplementation (monthly vitamin D tablet of 50,000 IU) or an identical placebo for 2 years.

As an extension of the VIDEO trial, 261 participants from Tasmania were contacted again after 3 years of completion of the VIDEO trial (i.e. 5 years from the initial date of taking either vitamin D or placebo (February to October 2017)). The flowchart of this study recruitment is shown in Supplementary Figure S1. Written informed consent was obtained from all participants in this study. This study was approved by the Tasmania Health and Medical Human Research Ethics Committee (No. H0016056).

### Serum vitamin D and inflammatory markers

Blood samples were collected at the local pathology centre at baseline and months 3, 24, and 60, and vitamin D status and inflammatory markers were assessed using methods consistent with the original VIDEO trial. The time of blood collection was documented to account for seasonal variation in vitamin D levels. The 25 (OH)D levels in serum was determined using the LIAISON® assay (Cardinal Health, Dublin, Ohio, USA), which is a direct competitive chemiluminescence immunoassay. The coefficients of variance of inter- and intra-assays were both 7.0%. Inflammatory markers (hs-CRP and IL-6) were analysed using enzyme-linked immunosorbent assay (ELISA) methods (IBL Inc. & Bio-Rad Laboratories Inc., respectively). The minimum level of detection was 0.003 ng/mL for IL-6 and was 0.178 mg/mL for hsCRP. The inter-assay and intra-assay coefficients of variances were < 10 and < 15% for all inflammatory biomarkers [31].

### Vitamin D maintainers and non-maintainers

Most of the previous studies and guidelines defined vitamin D deficiency as serum 25(OH)D concentrations below 50 nmol/L and vitamin D sufficiency as serum concentrations over 50 nmol/L [32, 33]. The vitamin D status of participants (total VIDEO trial participants irrespective of their treatment allocation) was classified into two groups based on the levels of 25(OH)D at 3-month, 24-month, and 60-month follow-up: (1) maintainer [serum 25(OH)D ≥ 50 nmol/L at 3-month, 24-month, and 60-month follow-up] and (2) non-maintainer [serum 25(OH)D levels < 50 nmol/L at any of the three follow-ups].

### Knee symptoms

A self-administered questionnaire, WOMAC, was employed to assess knee pain, function, and stiffness at baseline and months 3, 4, 6, 12, 24, and 60 [34]. A 100-mm visual analogue scale (VAS) questionnaire was also used to assess participants’ knee pain.

### Other questionnaires

Participants’ history of KS or TKR between month-24 and month-60 was documented by asking: “Have you had a knee surgery, including total or partial knee replacement, after completion of the VIDEO trial?” Regular use of vitamin D supplements (taking supplements at least five times per week for more than 9 months of the year) was also asked in the questionnaire. At baseline and months 3, 4, 6, 12, 24, and 60, psychological health or depression scores were assessed using the Patients Health Questionnaire-9 (PHQ-9) [35], and Quality of life (AQol-4D) was also assessed, from which utility values (0–1) were derived [36]. Physical activity was measured by the International Physical Activity Questionnaire (IPAQ) short version at baseline and months 24 and 60 [37].

### Sample size

The sample size calculations were based on the VAS pain change in the original VIDEO study (VAS pain in the placebo group was − 9.4 mm ± 21.1). We expected a VAS pain difference of 9 mm between the groups over 5 years (5.4 mm over 2 years). Assuming 80% power and two-sided significance level of 0.05, we need 88 participants per group (total of 176 participants). This is a feasible sample size even if we account for 70% response rate from Hobart centre (*n* = 261) participants of VIDEO study.

### Statistical methods

The independent students’ *t*-tests were used to compare differences in characteristics between two groups (vitamin D versus placebo; maintainer versus non-maintainer groups). Logistic regression was employed to compare the differences in the incidence of KS or TKR. The between-group difference in change in symptoms was analysed using repeated measures mixed-effects model with fixed terms for age, sex, body mass index (BMI), KS, physical activity [38], and treatment status or vitamin D maintainer status. The correlation within the repeated measures was addressed by using patient identification as a random effect. The effect of treatment or maintaining vitamin D sufficiency was estimated by the visit × treatment or maintaining vitamin D sufficiency status interaction terms.

We performed a subgroup analysis by excluding participants who underwent KS, using a similar analytical model above. KS as a treatment for knee OA often represents “joint death” or “overhaul”, and we performed subgroup analyses by removing participants who underwent KS. Surgery, including TKR occurred during the 24- and 60-month observational period, and their symptoms, utility, and depression scores were obtained at the 60-month follow-up only, which was after the surgery. Due to the observational nature of this study, removing KS patients represent a natural progression or trajectory of symptoms.

## Results

**Fig. 1:**
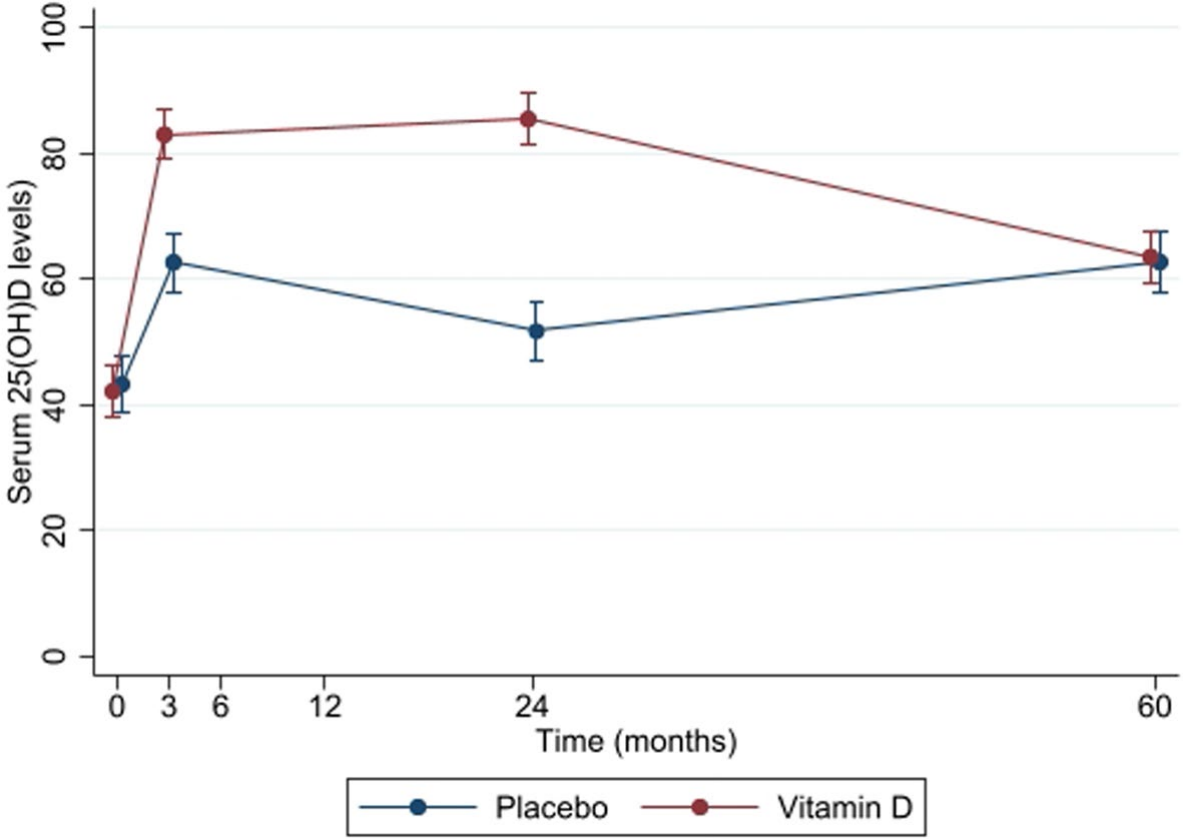
Change in serum 25(OH)D levels over 60 months by the original treatment group of the VIDEO trial. Vitamin D or placebo supplementation was completed at 24 months, and the change in vitamin D from 24 to 60 months was observational data (analysed using a repeated-measures mixed-effects model with fixed terms for seasons of blood collection, treatment, and participants’ identification as random effects)

A total of 173 participants (mean age 62.6, 48.6% women) were followed up 3 years after the cessation of original 2-year investigational treatment (vitamin D or placebo). The average level of 25(OH)D dropped in the vitamin D group (from 85.4 nmol/l to 63.6 nmol/l) and slightly increased in the placebo group (51.7 nmol/l to 62.8 nmol/l) 3 years after the cessation of the treatment (Fig. 1). Fifteen in the placebo group (including 11 TKR) and 16 in the vitamin D group (including 14 TKR) reported KS. There was no statistically significant difference in KS (OR 1.0 95% CI 0.5, 2.4) or TKR (OR 1.2 95% CI 0.5, 3.1) between vitamin D and placebo groups. Characteristics of the participants at 5 years follow-ups were shown in Table 1, changes from baseline and 24 months were shown in Supplementary Table S1, and characteristics of the participants based on self-reported knee surgery in the VIDEO trial at 60-month follow-up were shown in Supplementary Table S2.

**Table 1:** Characteristics of the participants based on the original allocation to vitamin D or placebo group in the VIDEO trial at 60-month follow-up

	Placebo (*n* = 81)	Vitamin D (*n* = 92)
Age, years	66.7 (10.1)	68.9 (6.9)
Female, *n* (%)	39 (48%)	45 (49%)
BMI, kg/m^2^	29.6 (12.1)	27.9 (8.6)
Knee surgery^a^, *n* (%)	15 (19%)	16 (17%)
Total knee replacement, *n* (%)	11 (14%)	14 (15%)
Vitamin D supplementation from 2014 to 2016		
No regular use, *n* (%)	45 (56%)	64 (70%)
Regular use for 1 or 2 years, *n* (%)	16 (20%)	11 (12%)
Regular use for 3 years, *n* (%)	20 (25%)	17 (18%)
Serum 25(OH)D levels, nmol/l	60.6 (19.4)	62.0 (18.4)
Serum hs-CRP, mg/ml	1.6 (1.3)	2.3 (2.8)
Serum IL6, ng/mL	1.5 (3.3)	1.3 (1.3)
WOMAC		
Pain (0–500)	91.7 (82.0)	101.5 (96.3)
Function (0–1700)	318.4 (287.9)	356.9 (351.2)
Stiffness (0–200)	45.9 (41.6)	42.0 (44.0)
Study knee VAS pain (0–100)	32.8 (25.6)	34.4 (29.2)
AQoL utility (0–1)	0.7 (0.2)	0.7 (0.2)
PHQ-9 (0–27)	2.8 (2.9)	3.1 (3.3)
No depression, *n* (%)	60 (74%)	68 (74%)
Mild depression, *n* (%)	18 (22%)	18 (20%)
Moderate to severe depression, *n* (%)	3 (4%)	6 (7%)
MET-min/week	3971.9 (3632.0)	3259.5 (3587.6)
Physical activity categories		
Low	18 (22.5%)	24 (26.1%)
Moderate	20 (25.0%)	31 (33.7%)
High	42 (52.5%)	37 (40.2%)

Data were shown as mean (SD) or otherwise stated

*BMI* body mass index, *hs-CRP* high sensitivity C Reactive protein, *TKR* total knee replacement, *WOMAC* Western Ontario and McMaster Universities Osteoarthritis Index, *AQoL* Assessment of Quality of Life, *PHQ9* Patient Health Questionnaire-9, *MET* metabolic equivalent of task

^a^Including TKR, partial knee replacement, bone graft, and partial meniscus removal, kneecap alignment, replacement of quadriceps tendon, and arthroscopy

There was no significant difference in change in WOMAC symptoms, knee VAS pain, depression scores, utility scores, and serum levels of IL6 and hs-CRP between vitamin D and placebo groups after 3 years of cessation of the clinical trial (Table 2). Among participants who reported no KS, there was a significant improvement in WOMAC function (*β*: − 83.7, 95% CI: − 167.3, 0.0) and depression scores (*β*: − 1.3, 95% CI: − 2.3, − 0.2) over 5 years in the treatment group compared to the placebo group.

**Table 2:** Change in clinical symptoms and inflammatory markers over 60 months based on the original allocation to vitamin D or placebo group in the VIDEO trial

	Placebo		Vitamin D		Between-group change
	*n*	Change	*n*	Change	
WOMAC pain	81	** − 25 (− 44.5, − 5.4)**	91	** − 26.3 (− 44.7, − 7.9)**	− 1.3 (− 28.4, 25.8)
No surgery	66	− 16.6 (− 37.3, 4.1)	75	** − 34.5 (− 53.9, − 15.2)**	− 17.9 (− 46.6, 10.8)
WOMAC function	81	** − 67.9 (− 126.4, − 9.4)**	91	** − 88.6 (− 143.6, − 33.5)**	− 20.7 (− 101.6, 60.3)
No surgery	66	− 34.6 (− 95, 25.9)	75	** − 118.3 (− 174.7, − 61.8)**	** − 83.7 (− 167.3, 0)**
WOMAC stiffness	81	** − 10.2 (− 18.9, − 1.4)**	91	** − 14 (− 22.2, − 5.8)**	− 3.9 (− 15.9, 8.2)
No surgery	66	− 5.5 (− 14.7, 3.6)	75	** − 17.3 (− 25.8, − 8.8)**	− 11.8 (− 24.4, 0.9)
Study knee VAS pain	81	** − 13.1 (− 18.7, − 7.5)**	92	** − 14.9 (− 20.2, − 9.7)**	− 1.9 (− 9.6, 5.9)
No surgery	66	** − 11.2 (− 17.3, − 5.1)**	76	** − 17 (− 22.7, − 11.3)**	− 5.8 (− 14.3, 2.6)
Utility	81	− 0.025 (− 0.054, 0.005)	90	** − 0.035 (− 0.063, − 0.007)**	− 0.01 (− 0.051, 0.031)
No surgery	66	− **0.037 (**− **0.07,** − **0.005)**	74	− 0.024 (− 0.054, 0.007)	0.014 (− 0.031, 0.058)
PHQ9	80	0.2 (− 0.4, 0.9)	92	− 0.5 (− 1.1, 0.1)	− 0.8 (− 1.7, 0.2)
No surgery	65	**0.8 (0, 1.5)**	76	− 0.5 (− 1.2, 0.2)	** − 1.3 (− 2.3, − 0.2)**
IL6	59	− 1.9 (− 6.5, 2.7)	80	** − 7.2 (− 11.3, − 3.1)**	− 5.3 (− 11.5, 0.9)
No surgery	48	− 1.7 (− 7.3, 3.8)	68	** − 8.1 (− 13.1, − 3.1)**	− 6.4 (− 13.8, 1.1)
hs-CRP	59	− 0.2 (− 0.8, 0.3)	80	− 0.1 (− 0.6, 0.3)	0.1 (− 0.7, 0.8)
No surgery	48	− 0.1 (− 0.7, 0.5)	68	− 0.1 (− 0.7, 0.4)	0 (− 0.8, 0.8)

*BMI*, body mass index; *hs-CRP*, high sensitivity C Reactive protein; *WOMAC*, Western Ontario and McMaster Universities Osteoarthritis Index; *AQoL*, Assessment of Quality of Life; *PHQ9*, Patient Health Questionnaire-9. The between-group difference in change in outcomes was analysed using repeated measures mixed-effects model with fixed terms for age, sex, BMI, KS, physical activity, and treatment status or vitamin D maintainer status. Bold figures denote statistical significance (*P* < 0.05)

Surgery, including TKR occurred during the 24- and 60-month observational period, and their symptoms, utility, and depression scores were obtained at the 60-month follow-up only, which was after the surgery

Seventy-nine participants maintained adequate vitamin D levels over 5 years, and 61 participants did not maintain adequate vitamin D levels over 5 years (Table 3 and Supplementary Table S3). Eleven in the non-maintainer group (including 10 TKR) and 11 in the maintainer group (including 8 TKR) reported KS. There was no statistically significant difference in KS (OR 0.7 95% CI 0.3, 1.8) or TKR (OR 0.6 95% CI 0.2, 1.6) between vitamin D maintainer and non-maintainer groups. There was no significant difference in change in WOMAC symptoms, knee VAS pain, depression scores, utility scores, and serum levels of IL6 and hs-CRP between vitamin D maintainer and non-maintainer groups after 3 years of cessation of the clinical trial (Table 4). Among participants who did not report KS, those who maintained adequate vitamin D levels over 5 years had significantly less WOMAC knee pain (*β*: − 33.9, 95% CI: − 65.7, − 2) and physical dysfunction (*β*: − 105.5, 95% CI: − 198.2, − 12.8) than participants with vitamin D deficiency over 5 years (Table 4).

**Table 3:** Characteristics of the participants among participants who maintained sufficient vitamin D levels and participants who did not maintain sufficient vitamin D levels at 60-month follow-up

	Vitamin D non-maintainer (*n* = 61)	Vitamin D maintainer (*n* = 79)
Age, years	68.3 (6.5)	68.2 (6.6)
Female, *n* (%)	29 (48%)	35 (44%)
BMI, kg/m^2^	29.6 (6.7)	27.8 (13.4)
Serum 25(OH)D levels, nmol/l	51.1 (18.8)	69.4 (14.4)
Serum hs-CRP, mg/ml	1.9 (1.6)	2.0 (2.7)
Serum IL6, pg/mL	1.5 (3.3)	1.3 (1.2)
Knee surgery^a^, *n* (%)	11 (18%)	11 (14%)
Total knee replacement, *n* (%)	10 (16%)	8 (10%)
Vitamin D supplementation from 2014 to 2016		
No regular use, *n* (%)	44 (72%)	48 (61%)
Regular use for 1 or 2 years, *n* (%)	8 (13%)	14 (18%)
Regular use for 3 years, *n* (%)	9 (15%)	17 (22%)
WOMAC		
Pain (0–500)	106.0 (96.5)	84.0 (79.2)
Function (0–1700)	353.6 (322.1)	300.1 (293.6)
Stiffness (0–200)	45.8 (43.6)	37.9 (36.7)
Study knee VAS pain (0–100)	33.4 (27.2)	30.4 (26.6)
AQoL utility (0–1)	0.7 (0.2)	0.7 (0.2)
PHQ-9 (0–27)	3.1 (3.1)	2.9 (3.0)
No depression, *n* (%)	46 (75%)	58 (73%)
Mild depression, *n* (%)	12 (20%)	17 (22%)
Moderate to severe depression, *n* (%)	3 (5%)	4 (5%)
MET-min/week	3383.1 (3662.7)	3810.3 (3741.4)
Physical activity categories		
Low	20 (32.8%)	14 (17.7%)
Moderate	17 (27.9%)	25 (31.6%)
High	24 (39.3%)	40 (50.6%)

Data were shown as mean (SD) or otherwise states

*BMI* body mass index, *hs-CRP* high sensitivity C Reactive protein, *TKR* total knee replacement, *WOMAC* Western Ontario and McMaster Universities Osteoarthritis Index, *AQoL* Assessment of Quality of Life, *PHQ9* Patient Health Questionnaire-9, *MET* metabolic equivalent of task

^a^Including TKR, partial knee replacement, bone graft, and partial meniscus removal, kneecap alignment, replacement of quadriceps tendon, and arthroscopy

**Table 4:** Change in clinical symptoms and serum inflammatory markers over 60 months in participants who maintained sufficient vitamin D levels and participants who did not maintain sufficient vitamin D levels

	Vitamin D non-maintainer		Vitamin D maintainer		Between-group change
	*n*	change	*n*	change	
WOMAC pain	60	− 15.3 (− 37.6, 7.1)	79	− **38.4 (**− **57.9,** − **18.9)**	− 23.1 (− 52.9, 6.6)
No surgery	49	− 10.5 (− 34.7, 13.6)	68	− **44.4 (**− **65,** − **23.8)**	− **33.9 (**− **65.7,** − **2)**
WOMAC function	60	− 42.2 (− 107.8, 23.4)	79	− **113 (**− **170.3,** − **55.7)**	− 70.8 (− 158.1, 16.5)
No surgery	49	− 22.4 (− 92.7, 48)	68	− **127.9 (**− **187.8,** − **68)**	− **105.5 (**− **198.2,** − **12.8)**
WOMAC stiffness	60	− **12.2 (**− **22,** − **2.5)**	79	− **15.4 (**− **23.9,** − **6.9)**	− 3.1 (− 16.1, 9.8)
No surgery	49	− 9.3 (− 19.7, 1.2)	68	− **16 (**− **24.8,** − **7.2)**	− 6.7 (− 20.4, 7)
Study knee VAS pain	61	− **13.1 (**− **19.4,** − **6.8)**	79	− **17.4 (**− **22.9,** − **11.8)**	− 4.2 (− 12.7, 4.2)
No surgery	50	− **12 (**− **19,** − **5.1)**	68	− **18.3 (**− **24.3,** − **12.3)**	− 6.3 (− 15.5, 2.9)
Utility	60	− 0.023 (− 0.057, 0.01)	78	− 0.013 (− 0.042, 0.016)	0.01 (− 0.034, 0.054)
No surgery	49	− 0.031 (− 0.068, 0.005)	67	− 0.01 (− 0.041, 0.021)	0.021 (− 0.027, 0.07)
PHQ9	61	0.2 (− 0.6, 0.9)	79	− 0.6 (− 1.3, 0.1)	− 0.8 (− 1.8, 0.3)
No surgery	50	0.5 (− 0.3, 1.4)	68	− 0.5 (− 1.2, 0.3)	− 1 (− 2.1, 0.1)
IL6	57	− **5 (**− **9.9,** − **0.1)**	73	− **5.1 (**− **9.4,** − **0.8)**	− 0.1 (− 6.6, 6.5)
No surgery	47	− 5.5 (− 11.4, 0.5)	62	− **5.5 (**− **10.6,** − **0.4)**	− 0.1 (− 7.9, 7.8)
hs-CRP	57	− 0.4 (− 0.9, 0.2)	73	0.1 (− 0.4, 0.5)	0.4 (− 0.3, 1.1)
No surgery	47	− 0.3 (− 0.8, 0.3)	62	− 0.1 (− 0.6, 0.4)	0.2 (− 0.6, 0.9)

*BMI* body mass index, *hs-CRP* high sensitivity C Reactive protein, *WOMAC* Western Ontario and McMaster Universities Osteoarthritis Index, *AQoL* Assessment of Quality of Life, *PHQ9* Patient Health Questionnaire-9. The between-group difference in change in outcomes was analysed using repeated measures mixed-effects model with fixed terms for age, sex, BMI, KS, physical activity, and treatment status or vitamin D maintainer status. Bold figures denote statistical significance (*P* < 0.05)

Surgery, including TKR occurred during the 24- and 60-month observational period, and their symptoms, utility, and depression scores were obtained at the 60-month follow-up only, which was after the surgery

## Discussion

To our best knowledge, this study is the first longitudinal study to investigate the long-term effects of vitamin D supplementation or maintaining sufficient serum vitamin D levels on symptoms, quality of life, psychological health, and inflammatory markers over 5 years in participants with knee OA. Two-year vitamin D supplementation or maintaining vitamin D sufficiency over 5 years was not significantly associated with improvement in symptoms of knee OA. However, among participants who did not report KS due to OA, 2-year vitamin D supplementation was associated with improved WOMAC function and depression scores over 5 years. Similarly, maintaining vitamin D sufficiency over 5 years was associated with improved WOMAC pain and function compared to participants who did not maintain sufficient vitamin D levels over 5 years.

Serum vitamin D levels regressed to the mean value of around 61.4 nmol/L after 3 years of the cessation of the VIDEO trial. However, this was significantly higher than the mean baseline vitamin D levels (42.7 nmol/L) before starting the trial. As expected, vitamin D levels decreased in the original vitamin D supplementation group and slightly increased in the placebo group. Data from the Dallas Heart Study suggests that vitamin D levels decreased after 7 years of follow-up despite an increase in vitamin D supplementation, suggesting a natural decline in the vitamin D levels [39].

The goal of grouping participants according to serum 25(OH)D levels is to evaluate the effectiveness of sufficient vitamin D levels on the outcomes, including WOMAC pain, function, quality of life, and depression status assessed by PHQ-9 scores, regardless of how the levels were achieved. Five-year follow-up of VIDEO RCT is an observational study and the original treatment and placebo groups were recontacted after 3 years of cessation the trial. Among participants who have 5-year follow-up 25(OH)D levels and were originally allocated to placebo group, 37% (22 out of 81) participants attained adequate serum 25(OH)D levels. This demonstrates that quantifying the proportion achieving adequate 25(OH)D levels in the placebo group provides important insights into explaining the negative results of vitamin D RCTs.

TKR is often considered as the ultimate outcome in RCTs, but their incidence is rare to practically conduct an appropriately powered RCT. Therefore, many RCTs in knee OA often link their patient data with joint replacement registries after the completion of the trial [40]. We found that 2-year vitamin D supplementation or maintaining sufficient vitamin D for 5 years was not associated with the incidence of KS or TKR. Previous studies have suggested that preoperative vitamin D status was associated with post-operative symptoms, but there is lack of evidence on the causal link between vitamin status and TKR [41]. Retrospective observational studies suggest that there is high prevalence of vitamin D deficiency among participants scheduled for total knee replacement [42, 43].

Vitamin D could play a role in the aetiology and maintenance of chronic pain states by exerting hormonal and immunological effects on pain [44]. Other potential mechanism includes vitamin D or vitamin D receptor that may influence pain sensitisation [45]. We found no association between vitamin D supplementation over 2 years and improvement in knee symptoms over 5 years. Similarly, there was no association between maintaining sufficient vitamin D over 5 years and improvement in knee symptoms over 5 years.

Our 5-year observational cohort had participants who underwent KS or TKR (occurred between 24- and 60-month observational period only) and their symptoms, utility, and depression scores were obtained at 60 months (after the surgery or TKR). The original VIDEO trial excluded TKR at any stage of the trial. Therefore, we conducted a sub-group analysis by excluding participants (excluding “joint death” or “overhaul”) who underwent surgery to represent a natural progression of OA. We found that among participants who did not report KS, vitamin D supplementation over 2 years was associated with a modest improvement in WOMAC function (*β* − 83.7; 95% CI, − 167.3, 0.0) but not WOMAC pain over 5 years. This is consistent with the 2-year results of the original VIDEO trial, where we reported that the 2-year supplementation was associated with a significant improvement in WOMAC function (*β* − 72.9; 95% CI, − 126.4, − 19.4) but not WOMAC pain over 2 years among the whole participants [18].

Similarly, maintaining sufficient vitamin D over 5 years was associated with a modest but statistically significant improvement in WOMAC knee pain (*β* − 33.9; 95% CI, − 65.7, − 2) and WOMAC function (*β* − 105.5; 95% CI, − 198.2, − 12.8) over 5 years. The effect size on pain and function is larger than the post hoc results from the original VIDEO trial, where we reported that maintaining sufficient vitamin D levels over 2 years was associated with improvement in WOMAC pain (*β* − 15.4; 95% CI, − 49.9, 19.2) and WOMAC dysfunction (*β* − 94.2; 95% CI, − 183.8, − 4.5) over 2 years [19]. The effect size we observed may not be clinically significant but larger than the results we observed over 2 years [46]. Our results indicate that vitamin D supplementation or maintaining sufficient vitamin D levels may have a long-term modest effect on knee OA, due to either (i) longer cumulative exposure beyond 2 years or (ii) lagged effects of vitamin D exposure up to 2 years, or a combination of the two.

We have previously reported that vitamin D supplementation over 24 months may have a beneficial effect on depressive symptoms (*β* − 0.66, 95% CI: − 1.22 to − 0.11) than placebo in knee OA patients [47]. In this 5-year follow-up study, we found that among participants who did not report KS, vitamin D supplementation was associated with a statistically significant reduction in depressive symptoms (*β* − 1.3; 95% CI, − 2.3, -0.2). The effect size in this study was larger than the post hoc analysis of the original 2-year trial, indicating the potential long-term effects of vitamin D supplementation. A systematic review of observational studies has reported that low vitamin D concentration is associated with depression [48]. However, a meta-analysis of RCTs assessing the efficacy of vitamin D supplementation on depression in the general, obese, and other diseased populations has not found a significant beneficial effect [49].

A strength of this study is the long-term follow-up of a vitamin D randomised clinical trial population. However, there are limitations to this study. We did not follow-up all participants from Tasmania who were originally enrolled in the VIDEO study (lost to follow-up of 34% of the participants (88 out of 261)). However, the baseline characteristics of the participants who completed the 5-year follow-up were comparable to those who did not complete the follow-up, except that the physical activity was higher than those lost to follow-up (Supplementary Table S4). Loss to follow-up bias may still exist because we do not have further information on the participants who lost to follow-up after 2 years.

In conclusion, vitamin D supplementation over 2 years or maintaining vitamin D sufficiency for 5 years was not associated with significant differences in change in knee symptoms over 5 years. However, among participants who did not report KS, 2-year vitamin D supplementation was linked to a modest improvement in knee dysfunction and depression levels. Similarly, knee OA patients maintaining sufficient serum vitamin D levels over 5 years was associated with a modest improvement in knee pain and physical dysfunction than those who did not maintain adequate vitamin D levels, suggesting a potential beneficial effect of maintaining sufficient serum vitamin D for knee OA.

### Supplementary Information

**Additional file 1:** **Supplementary Figure S1.** Flowchart of VIDEO five-year follow-up study. **Supplementary Table S1.** Change in WOMAC symptoms and Vitamin D levels of the participants based on the original allocation to vitamin D or placebo group in the VIDEO trial over 5 years. **Supplementary Table S2.** Characteristics of the participants based on self-reported knee surgery in the VIDEO trial at 60-month follow-up. **Supplementary Table S3.** Change in WOMAC symptoms and Vitamin D levels among participants who maintained sufficient vitamin D levels and participants who did not maintain sufficient vitamin D levels over 5 years. **Supplementary Table S4.** Baseline characteristics of the participants who completed the five-year follow-up and lost to follow-up.

## Data Availability

The datasets generated and/or analysed during the current study are not publicly available due to institute policy but are available from the corresponding author on reasonable request.

## Abbreviations

OA: Osteoarthritis
VIDEO: Vitamin D Effects on Osteoarthritis trial
RCT: Randomised clinical trials
NSAIDs: Non-steroidal anti-inflammatory drugs
25(OH)D: 25-Hydroxy-vitamin D
JSN: Joint space narrowing
WOMAC: The Western Ontario and McMaster Universities Osteoarthritis Index
ELISA: Enzyme-linked immunosorbent assay
VAS: Visual analogue scale
KS: Knee surgery
TKR: Total knee replacement
PHQ-9: Patients Health Questionnaire-9
AQol-4D: Quality of life
IPAQ: International Physical Activity Questionnaire
BMI: Body mass index
