# Supplementary material for: Long-term effects of vitamin D supplementation and maintaining sufficient vitamin D on knee osteoarthritis over 5 years
Source: Arthritis Res Ther. 2023 Sep 23;25:178. doi: 10.1186/s13075-023-03167-8 (PMC10517449; doi:10.1186/s13075-023-03167-8)
Supplement: Supplementary file 1 — Additional file 1: Supplementary Figure S1. Flowchart of VIDEO five-year follow-up study. Supplementary Table S1. Change in WOMAC symptoms and Vitamin D levels of the participants based on the original allocation to vitamin D or placebo group in the VIDEO trial over 5 years. Supplementary Table S2. Characteristics of the participants based on self-reported knee surgery in the VIDEO trial at 60-month follow-up. Supplementary Table S3. Change in WOMAC symptoms and Vitamin D levels among participants who maintained sufficient vitamin D levels and participants who did not maintain sufficient vitamin D levels over 5 years. Supplementary Table S4. Baseline characteristics of the participants who completed the five-year follow-up and lost to follow-up. [file 13075_2023_3167_MOESM1_ESM.docx]

**Supplementary materials to:**

**Long-term effects of vitamin D supplementation and maintaining sufficient vitamin D on knee osteoarthritis over five years**

Zhiqiang Wang^a,b^, Zhaohua Zhu^a^, Feng Pan^b^, Shuang Zheng^c^, Venkat Parameswaran^d^, Leigh Blizzard^b^, Changhai Ding^a,b,e*^, Benny Antony^b*^

^a^ Clinical Research Centre, Zhujiang Hospital, Southern Medical University, Guangzhou, China.

^b^ Menzies Institute for Medical Research, University of Tasmania, Hobart, Australia.

^c^ Department of Rheumatology and Immunology, Arthritis Research Institute, the First Affiliated Hospital of Anhui Medical University, Hefei, China.

^d^ Department of Endocrinology, Royal Hobart Hospital, Hobart, Tasmania, 7000 Australia.

^e^ Department of Epidemiology and Preventive Medicine, Monash University, Melbourne, Australia.

*Correspondence to Benny Antony, Menzies Institute for Medical Research, University of Tasmania, Australia, email: [Benny.EathakkattuAntony@utas.edu.au](mailto:Benny.EathakkattuAntony@utas.edu.au); and Changhai Ding, Clinical Research Centre, Zhujiang Hospital, Southern Medical University, Guangzhou, China Email: [changhai.ding@utas.edu.au](mailto:changhai.ding@utas.edu.au)

Supplementary Figure S1. Flowchart of VIDEO five-year follow-up study.


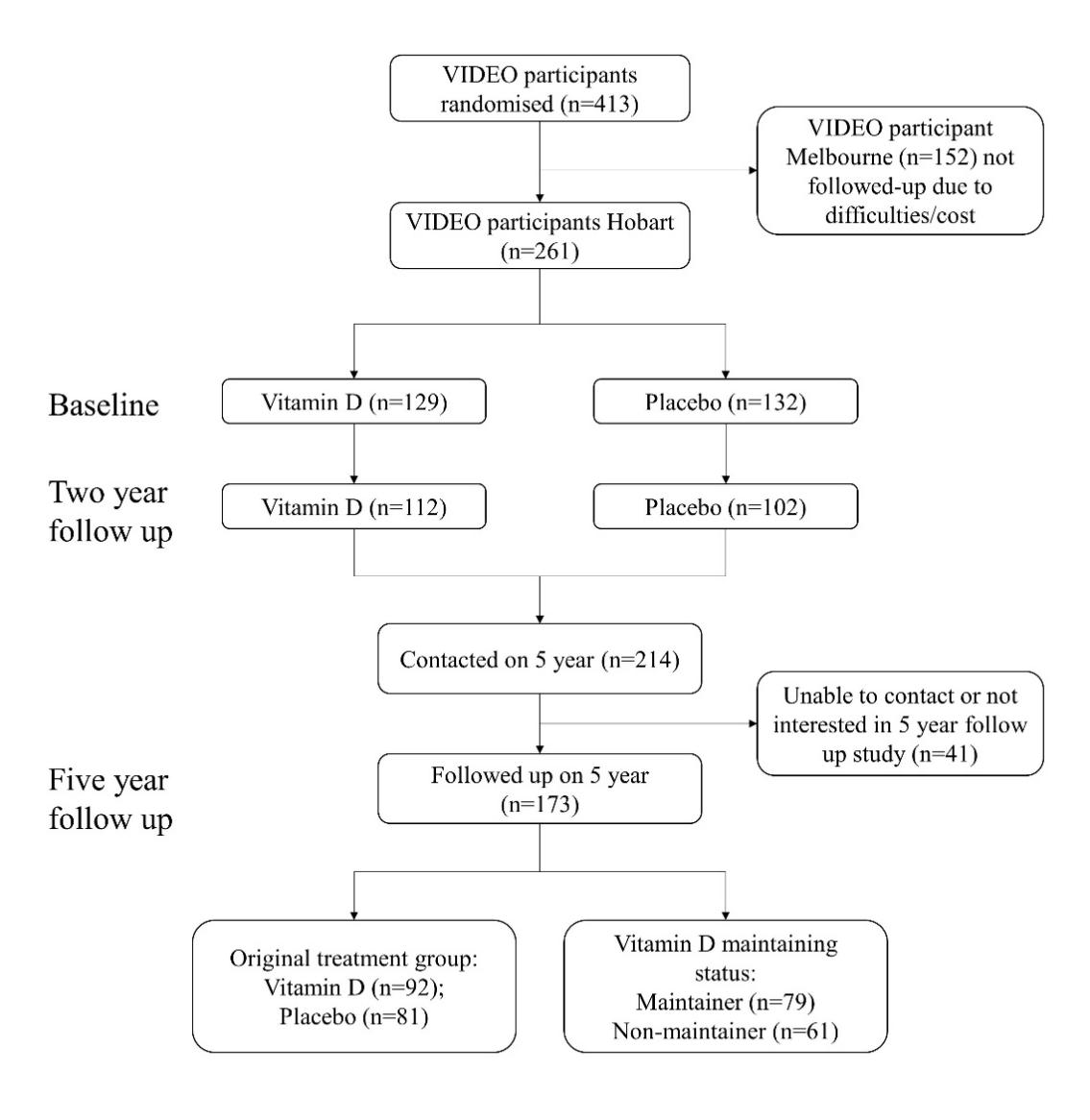


Supplementary Table S1. Change in WOMAC symptoms and Vitamin D levels of the participants based on the original allocation to vitamin D or placebo group in the VIDEO trial over five years

|  | Placebo (n=81) | Vitamin D (n=92) |
| --- | --- | --- |
| **Change from 24 months** | | |
| Serum 25(OH)D levels, nmol/l | 6.9 (23.6) | -24.9 (19.4) |
| Serum hs-CRP, mg/ml | -0.1 (1.5) | -0.3 (2.6) |
| Serum IL6, ng/mL | -0.6 (6.3) | -3.6 (13.5) |
| WOMAC |  |  |
| Pain (0-500) | 20.1 (86.9) | 22.5 (97.6) |
| Function (0-1700) | 38.1 (235.3) | 84.7 (308.0) |
| Stiffness (0-200) | 9.7 (38.6) | 4.3 (38.6) |
| **Change from baseline** |  |  |
| Serum 25(OH)D levels, nmol/l | 17.3 (20.5) | 19.9 (20.9) |
| Serum hs-CRP, mg/ml | -0.0 (1.6) | -0.2 (2.5) |
| Serum IL6, ng/mL | -1.5 (9.8) | -8.4 (29.6) |
| WOMAC |  |  |
| Pain (0-500) | -27.9 (84.7) | -23.4 (102.7) |
| Function (0-1700) | -76.4 (282.7) | -80.6 (370.9) |
| Stiffness (0-200) | -11.7 (42.9) | -13.2 (46.2) |

Data were shown as mean (SD) or otherwise states. BMI, body mass index; hs-CRP, high sensitivity C Reactive protein; TKR, total knee replacement; WOMAC, Western Ontario and McMaster Universities Osteoarthritis Index.

Supplementary Table S2. Characteristics of the participants based on self-reported knee surgery in the VIDEO trial at 60-month follow-up.

|  | No surgery (n=142) | Surgery (n=31) |
| --- | --- | --- |
| Age, years | 67.4 (8.8) | 70.2 (6.9) |
| Female, n (%) | 69 (48.6%) | 15 (48.4%) |
| BMI, kg/m^2^ | 27.9 (10.8) | 32.3 (7.3) |
| Vitamin D supplementation from 2014 to 2016 | | |
| No regular use, n (%) | 89 (62.7%) | 20 (64.5%) |
| Regular use for one or two years, n (%) | 24 (16.9%) | 3 (9.7%) |
| Regular use for three years, n (%) | 29 (20.4%) | 8 (25.8%) |
| Serum 25(OH)D levels, nmol/l | 61.9 (18.6) | 58.6 (20.0) |
| Serum hs-CRP, mg/ml | 1.9 (2.2) | 2.3 (2.6) |
| Serum IL6, ng/mL | 1.4 (2.5) | 1.2 (1.1) |
| WOMAC | | |
| Pain (0-500) | 92.9 (85.0) | 115.1 (109.0) |
| Function (0-1700) | 320.6 (314.3) | 422.4 (352.9) |
| Stiffness (0-200) | 42.6 (42.4) | 49.6 (44.9) |
| Study knee VAS pain (0-100) | 33.0 (26.9) | 35.9 (31.0) |
| AQoL utility (0-1) | 0.7 (0.2) | 0.7 (0.2) |
| PHQ-9 (0-27) |  |  |
| No depression, n (%) | 101 (71.1%) | 27 (87.1%) |
| Mild depression, n (%) | 32 (22.5%) | 4 (12.9%) |
| Moderate to severe depression, n (%) | 9 (6.3%) | 0 (0.0%) |
| MET-min/week | 3709.7 (3706.1) | 3058.7 (3167.6) |
| Physical activity categories |  |  |
| Low | 32 (22.7%) | 10 (32.3%) |
| Moderate | 44 (31.2%) | 7 (22.6%) |
| High | 65 (46.1%) | 14 (45.2%) |

Data were shown as mean (SD) or otherwise stated. BMI, body mass index; hs-CRP, high sensitivity C Reactive protein; WOMAC, Western Ontario and McMaster Universities Osteoarthritis Index; AQoL, Assessment of Quality of Life; PHQ9, Patient Health Questionnaire-9; MET, metabolic equivalent of task.

Supplementary Table S3. Change in WOMAC symptoms and Vitamin D levels among participants who maintained sufficient vitamin D levels and participants who did not maintain sufficient vitamin D levels over five years

|  | Vitamin D non-maintainer (n= 61) | Vitamin D maintainer (n= 79) |
| --- | --- | --- |
| **Change from 24 months** | | |
| Serum 25(OH)D levels, nmol/l | -9.4 (34.2) | -12.7 (18.5) |
| Serum hs-CRP, mg/ml | -0.6 (2.4) | 0.0 (2.0) |
| Serum IL6, ng/mL | -1.4 (6.5) | -3.0 (13.7) |
| WOMAC |  |  |
| Pain (0-500) | 25.8 (95.2) | 16.5 (91.3) |
| Function (0-1700) | 59.5 (270.6) | 54.4 (288.7) |
| Stiffness (0-200) | 7.6 (37.0) | 4.8 (40.3) |
| **Change from baseline** |  |  |
| Serum 25(OH)D levels, nmol/l | 9.8 (19.7) | 25.6 (18.8) |
| Serum hs-CRP, mg/ml | -0.3 (2.4) | 0.0 (2.0) |
| Serum IL6, ng/mL | -5.0 (26.5) | -5.9 (21.3) |
| WOMAC |  |  |
| Pain (0-500) | -14.6 (107.0) | -38.8 (82.0) |
| Function (0-1700) | -44.3 (323.7) | -113.0 (315.4) |
| Stiffness (0-200) | -13.1 (43.6) | -15.3 (40.0) |

Data were shown as mean (SD) or otherwise states. BMI, body mass index; hs-CRP, high sensitivity C Reactive protein; TKR, total knee replacement; WOMAC, Western Ontario and McMaster Universities Osteoarthritis Index.

Supplementary Table S4. Baseline characteristics of the participants who completed the five-year follow-up and lost to follow-up.

|  | Five-year follow-up (n= 173) | Lost to follow-up (n= 88) |
| --- | --- | --- |
| Age, years | 62.6 (6.9) | 63.8 (7.9) |
| Female, n (%) | 84 (48.6%) | 45 (51.1%) |
| BMI, kg/m^2^ | 29.6 (4.7) | 29.7 (5.4) |
| Serum 25(OH)D levels, nmol/l | 43.4 (11.9) | 43.0 (12.6) |
| Serum hs-CRP, mg/ml | 2.5 (3.6) | 2.3 (3.7) |
| Serum IL6, pg/mL | 6.0 (19.8) | 7.7 (20.6) |
| WOMAC |  |  |
| Pain (0-500) | 121.8 (85.3) | 128.4 (82.4) |
| Function (0-1700) | 414.9 (303.9) | 458.2 (268.3) |
| Stiffness (0-200) | 55.8 (40.8) | 60.4 (40.7) |
| Study knee VAS pain (0-100) | 51.1 (17.8) | 49.7 (17.1) |
| AQoL utility (0-1) | 0.7 (0.2) | 0.7 (0.2) |
| PHQ-9 (0-27) | 3.1 (3.7) | 3.2 (4.4) |
| No depression, n (%) | 129 (75.0%) | 64 (74.4%) |
| Mild depression, n (%) | 29 (16.9%) | 16 (18.6%) |
| Moderate to severe depression, n (%) | 14 (8.1%) | 6 (7.0%) |
| MET-min/week | 3795.0 (3338.8) | 2974.3 (3770.6) |
| Physical activity categories |  |  |
| Low | 24 (14.4%) | 20 (24.4%) |
| Moderate | 64 (38.3%) | 33 (40.2%) |
| High | 79 (47.3%) | 29 (35.4%) |

Data were shown as mean (SD) or otherwise states. BMI, body mass index; hs-CRP, high sensitivity C Reactive protein; TKR, total knee replacement; WOMAC, Western Ontario and McMaster Universities Osteoarthritis Index; AQoL, Assessment of Quality of Life; PHQ9, Patient Health Questionnaire-9; MET, metabolic equivalent of task.

**
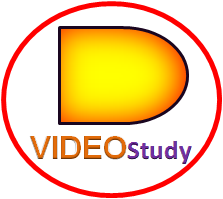
**
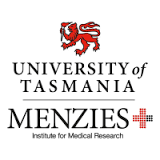


**VIDEO Follow-up Study**

**Long-term Effects of Vitamin D Supplementation on Knee Osteoarthritis**

**Scientific title**

**Does vitamin D supplementation over 2 years have long-term effects on knee osteoarthritis over 3 years after supplementation?**

**Sponsor Institution**

Menzies Institute for Medical Research

**Chief Investigators**

Prof Changhai Ding

Dr Benny Antony

**Contact details:**

Prof Changhai Ding

Dr Benny Antony

Menzies Institute for Medical Research

University of Tasmania

17 Liverpool Street, Hobart TAS 7000

Phone – (03) 62267730 Fax – (03) 62267704

**1. Background**

Osteoarthritis (OA) is characterized by gradual loss of articular cartilage and changes of other joint structures (i.e., subchondral bone and meniscus) leading, eventually, to total joint replacement. It is the most common joint disorder in the world and in Western populations is one of the most frequent causes of pain, loss of function and disability in adults [1]. Approximately 25% of people 55 years of age or older have had knee pain on most days in a month in the past year. Of these about half have radiographic knee OA and are considered to have symptomatic OA [1]. There is clearly an urgent need for research that investigates innovative and cost-effective approaches to slow the progression of OA.

Vitamin D deficiency [defined as serum level of 25-hydroxy-vitamin D(25-(OH)D) < 50 nmol/l] is very common in older people. In Australia, up to 80% of women and 70% of men living in hostels or nursing homes were deficient in vitamin D. High rates of vitamin D deficiency have been reported in all sectors of the community especially in Tasmania where up to 51% of women and 39% of men aged 50-79 years were deficient.

***Pathophysiological processes of vitamin D in normal and OA joints***

Vitamin D may have direct effects on chondrocytes in osteoarthritic cartilage. Vitamin D receptors (VDR) have been demonstrated in human articular chondrocytes (HAC) of osteoarthritic cartilage, especially the superficial zone [2]. VDR expression by HAC is often associated with sites where matrix metalloproteinases (MMPs) expression is prevalent, and 1α-25(OH)_2_D_3_ contributes to the regulation of MMP and PGE_2_ production by HAC in osteoarthritic cartilage [2]. Thus, articular cartilage, including cartilage affected by OA, seems to be sensitive to the effects of vitamin D.

Vitamin D may also exert an effect on OA through bone. During bone growth, vitamin D regulates the transition from growth plate cartilage to bone [3]. Chronic vitamin D inadequacy in adults has adverse effects on calcium metabolism, osteoblast activity, matrix ossification, and bone density, resulting in increased bone turnover and enhanced bone loss [4]. Thus vitamin D deficiency could impair the ability of bone to respond optimally to pathophysiological processes in OA, and predispose to disease progression.

In summary, there is potential for vitamin D supplementation to have beneficial effects on OA through its direct action on cartilage, and/or its effects on bone and muscle health.

***VIDEO study***

Vitamin D deficiency is linked with knee OA and knee pain. Recently, we conducted a clinical trial to examine if vitamin D supplementation over 2 years could improve the structural and clinical progression of knee OA. Vitamin D Effects on Osteoarthritis (VIDEO) study was the first randomised controlled trials to explore the effects of the vitamin D supplementation on knee structural changes (cartilage and bone) , knee pain and limb muscle strength assessment in knee OA patients with vitamin D deficiency. It was supported by a project grant from National Health & Medical Research Council (NHMRC ID 605501).

We found that vitamin D supplementation over 2 years did not significantly reduce WOMAC (a pain measuring scale)- measured knee pain, but significantly reduced VAS (another pain measuring scale)- measured knee pain and improved physical function compared to the placebo in patients with knee OA and moderate vitamin D deficiency [5]. There were more OMERACT-OARSI (Outcome Measures in Rheumatology Clinical trials- Osteoarthritis Research Society International) responders in the vitamin D group than the placebo group (35% vs. 25%, p = 0.029) and vitamin D group significantly reduce the increase in joint effusion (local inflammation) compared to the placebo. These results were reported as oral presentations at several prestigious international conferences, and a paper has been published in JAMA[5]. Further post-hoc analyses showed that vitamin D supplementation could reduce depression and food pain in knee OA.

Whilst these are promising results, the effects were modest and only significant at 2 years. These participants may have a prolonged beneficial effect from vitamin D supplementation for more than 2 years [6]. Currently, it has been 5 years since our initial observation.

# 2. Aims

- To investigate the effect of vitamin D supplementation compared to placebo over 2 years on knee pain, stiffness, physical function and incidence of total knee replacement over 5 years in patients with knee OA.
- To investigate the effect of vitamin D supplementation compared to placebo over 2 years on quality of life, physical health and psychological health over 5 years in patients with knee OA.
- To investigate the effect of vitamin D supplementation compared to placebo over 2 years on foot pain and back pain over 5 years in patients with knee OA.
- To investigate the effect of vitamin D supplementation compared to placebo over 2 years on inflammatory markers (measured using hs-CRP and IL-6) for up to 3 years following trial completion.
- To describe the changes in levels of inflammatory markers and vitamin D status with changes in knee pain, physical function, quality of life and total knee replacement in patients with knee OA.

# 3. Methods

*3.1 Study design*

VIDEO study was a randomised, placebo-controlled double-blind clinical trial. The current study is a follow-up study of VIDEO.

413 subjects with symptomatic knee OA (261 patients from Tasmania and 152 in Melbourne) were recruited by using a combined strategy, including collaboration with general practitioners, specialist rheumatologists, and orthopaedic surgeons, as well as advertising through local media. Patients with knee OA were randomised to vitamin D supplementation group or placebo group and they took monthly vitamin D (50,000 IU) or placebo for 2 years. 209 participants received vitamin D and 204 received placebo over 2 years.

This study is an extension of VIDEO trial. 260 participants from Tasmania will be contacted again after 3 years of completion (ie 5 years from the initial date of taking either vitamin D or placebo) and the questionnaires will be sent to them by mails. They will be called over telephone and help them filling in information including their current knee pain, physical function, quality of life, foot and back pain, depression, and if they have had a total knee replacement recently. The other information about the possible confounders (factors that influence their knee pain such as medication history, other diseases etc.) will also be collected. Blood will be collected at the local pathology center and vitamin D status and inflammatory markers will be assessed.

*3.2 Inclusion criteria:*

1. All participants who are original participants of VIDEO study in Tasmania.

*3.3 Exclusion criteria:*

1. Patients who are not contactable.

**4. Previous measurements in VIDEO study and new measurements at 5 year Follow-up**

|  | **Pre-screening** | **0 months** | **3 months** | **6 months** | **12 months** | **24 months** | **5yr Follow-up** |
| --- | --- | --- | --- | --- | --- | --- | --- |
| MRI |  | ✓ |  |  |  | ✓ |  |
| Knee pain (VAS) | ✓ | ✓ | ✓ | ✓ | ✓ | ✓ | ✓ |
| Knee radiograph | ✓ |  |  |  |  |  |  |
| Serum 25-(OH)D | ✓ |  | ✓ |  |  | ✓ | ✓ |
| Serum calcium, phosphate, Creatinine | ✓ |  | ✓ |  |  |  |  |
| Core musculature measure |  | ✓ |  |  | ✓ | ✓ |  |
| Lower limb muscle strength |  | ✓ | ✓ | ✓ | ✓ | ✓ |  |
| Hand grip strength |  | ✓ | ✓ | ✓ | ✓ | ✓ |  |
| Weight |  | ✓ | ✓ | ✓ | ✓ | ✓ | ✓ |
| Height |  | ✓ |  |  |  | ✓ | ✓ |
| Skin fold |  | ✓ |  |  |  | ✓ |  |
| Girth measurements |  | ✓ |  |  |  | ✓ |  |
| Upper arm pressure |  | ✓ | ✓ | ✓ | ✓ | ✓ |  |
| Central blood pressure |  | ✓ |  |  |  | ✓ |  |
| Ambulatory blood pressure |  | ✓ |  |  |  | ✓ |  |
| Arterial stiffness |  | ✓ |  |  |  | ✓ |  |
| Physical activity (IPAQ) |  | ✓ |  |  |  | ✓ | ✓ |
| Medications | ✓ | ✓ | ✓ | ✓ | ✓ | ✓ | ✓ |
| WOMAC |  | ✓ | ✓ | ✓ | ✓ | ✓ | ✓ |
| Sun exposure |  | ✓ |  | ✓ | ✓ | ✓ | ✓ |
| Low back pain |  | ✓ | ✓ | ✓ | ✓ | ✓ | ✓ |
| Foot pain |  | ✓ | ✓ | ✓ | ✓ | ✓ | ✓ |
| Depression |  | ✓ | ✓ | ✓ | ✓ | ✓ | ✓ |
| Quality of life |  | ✓ | ✓ | ✓ | ✓ | ✓ | ✓ |
| Cigarette smoking |  | ✓ |  |  |  | ✓ | ✓ |
| Previous knee injury, occupation |  | ✓ |  |  |  | ✓ | ✓ |
| Serum Inflammatory markers |  | ✓ |  |  |  | ✓ | ✓ |
| Adverse events |  | ✓ | ✓ | ✓ | ✓ | ✓ |  |

**4.1 Knee pain**

Knee pain, stiffness and function later will be assessed by the Western Ontario and McMaster Universities Osteoarthritis Index (WOMAC), a self-administered questionnaire [7]. It will also be assessed using a 100-mm visual analog scale (VAS).

**4.2 Serum vitamin D and inflammatory markers**

Serum samples will be collected at the local pathology centers and were treated initially with acetronitrile to rapidly extract 25-hydroxyvitamin D [25-(OH)D] and other hydroxylated metabolites. 25-(OH)D was then assayed utilising a Liquid Phase radioimmunoassay (Immunodiagnostics Systems Ltd, Boldon, Tyne & Wear, UK). Hs-CRP and IL-6 will be assessed using routine biochemical methods.

**4.3 Physical activity**

Physical activity will be measured by the International Physical Activity Questionnaire (IPAQ) [8] short version.

**4.4 Other Questionnaires**

Depression (using Patients Health Questionnaire-9, PHQ-9), cigarette smoking status (currently smoking, level of smoking, ever smoked, when gave up), previous knee injury and occupation, low back pain, foot pain and quality of life will be assessed by questionnaires.

**5. Informed consent**

5.1 Consent Form

Before obtaining consent from each participant, he /she must be informed of the objectives, benefits, risks and requirements of the study. An information sheet should be given to every participant prior to recruitment.

Participants will be giving their own consent after having read all content of the information sheet and consent form. Participant and investigator each retain a copy of the signed consent form.

5.2 Obtaining Consent

The Investigator, or a person designated by the Investigator, and under the Investigator's responsibility, should fully inform the participant of all pertinent aspects of the VIDEO Follow-up study including the written participant information sheet. All participants should be informed to the fullest extent possible about the study, in language and terms they are able to understand.

Participants are expected to be giving their own consents by reading content in the information sheet and consent form and giving their signatures by themselves. In those who might have vision impairment, a research officer may read out content and ensure a thoroughly understanding of all content before asking for signature.

- Prior to a subject’s participation in the study, the written Informed Consent Form should be signed, name filled in and personally dated by the participant or by the participant’s legally acceptable representative, and by the person who conducted the informed consent discussion.
- A copy of the signed and dated written Informed Consent Form will be provided to the participant. The original consent is to be stored in the participant’s individual study file, held by the investigator.
- The Participant information Sheet and Consent Form used for obtaining the participant's informed consent must be the current version that has been reviewed and approved by the appropriate Ethics Committee.

**6. Analyses:**

**6.1. Rationale:**

Primary outcomes of the original VIDEO trial had a statistically non-significant improvement; however, participants in the vitamin D group had statistically significant improvements in visual analog scale (VAS) knee pain, WOMAC function, total WOMAC score, increase in bone marrow lesions (BMLs) and joint effusion. There were 90 (50%) participants in the vitamin D group and 65 (39%) in the placebo group (P = 0.03) who showed at least a 50% improvement in WOMAC pain score. There were more OMERACT-OARSI (Outcome Measures in Rheumatology Clinical trials- Osteoarthritis Research Society International) responders in the vitamin D group than the placebo group (35% vs. 25%, p = 0.03).

The effects vitamin D supplementation on outcomes including VAS pain and function were consistent and are comparable to those from an existing treatment such as acetaminophen, non-steroidal anti-inflammatory drugs or glucosamine. Given that vitamin D supplementation is safe, cheap and convenient (once monthly) therapy, a modest benefit may be more clinically meaningful than existing therapies which have important limitations, such as poor safety and tolerability or limited efficacy. Moreover, there was continued improvement in the WOMAC pain score beyond 12 months in the vitamin D group, whereas the scores were unchanged in the placebo group (Figure 1). Although it is impossible to predict what the results would have been if the follow-up is longer, the possibility that vitamin D has sustained effects cannot be ruled out. Several studies have reported that vitamin D supplementation has sustained effects on bone mass and its outcomes.


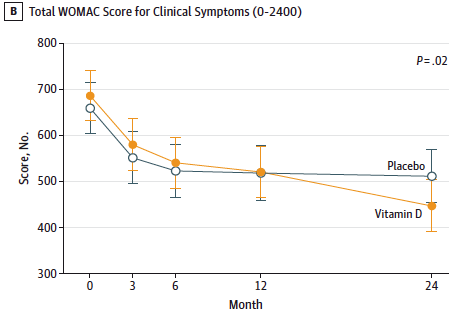


Figure 1: Comparison Between Vitamin D Group and Placebo Group on Change in Clinical Symptoms assessed by the total WOMAC score. (Please note that the change was most visible after 1 year of treatment)

**6.2 Power Calculation:**

Change in VAS pain in placebo group was -9.4 ± 21.1 in VIDEO study. We expect the difference between groups over 5 years will be 9 mm (5.4 mm over 2 years), and then 88 participants per group will be required. Therefore, around 176 participants will be required to detect the differences between groups. This is reasonable because it is assumed that 30% participants from 261 in VIDEO study will lose to follow-up over 5 years.

Current knee pain, stiffness and dysfunction and prevalence of total knee replacement will be analysed as main outcomes and will be compared between vitamin D and placebo groups. Further more, using mixed effects model, we will compare the tracking (slope) of knee pain, function and stiffness between vitamin D and placebo groups.

Current serum inflammatory markers (hs-CRP and IL-6) and vitamin D status will be compared between vitamin D and placebo groups. Changes in levels of inflammatory markers and vitamin D status will be compared with changes in knee pain, physical function, quality of life and total knee replacement.

Other variables including the current vitamin D status will be used for adjustments in the statistical models.

# 6. References

1. Felson DT: **Clinical practice. Osteoarthritis of the knee**. *N Engl J Med* 2006, **354**(8):841-848.

2. Tetlow LC, Woolley DE: **Expression of vitamin D receptors and matrix metalloproteinases in osteoarthritic cartilage and human articular chondrocytes in vitro**. *Osteoarthritis Cartilage* 2001, **9**(5):423-431.

3. McAlindon TE: **Nutraceuticals: do they work and when should we use them?** *Best Pract Res Clin Rheumatol* 2006, **20**(1):99-115.

4. Holick MF: **High prevalence of vitamin D inadequacy and implications for health**. *Mayo Clin Proc* 2006, **81**(3):353-373.

5. Jin X, Jones G, Cicuttini F, Wluka A, Zhu Z, Han W, Antony B, Wang X, Winzenberg T, Blizzard L *et al*: **Effect of Vitamin D Supplementation on Tibial Cartilage Volume and Knee Pain Among Patients With Symptomatic Knee Osteoarthritis: A Randomized Clinical Trial**. *JAMA* 2016, **315**(10):1005-1013.

6. Jin X, Ding C: **Vitamin D Supplementation and Progression of Knee Osteoarthritis--Reply**. *Jama* 2016, **316**(3):348-349.

7. Bellamy N, Buchanan WW, Goldsmith CH, Campbell J, Stitt LW: **Validation study of WOMAC: a health status instrument for measuring clinically important patient relevant outcomes to antirheumatic drug therapy in patients with osteoarthritis of the hip or knee**. *J Rheumatol* 1988, **15**(12):1833-1840.

8. Craig CL, Marshall AL, Sjostrom M, Bauman AE, Booth ML, Ainsworth BE, Pratt M, Ekelund U, Yngve A, Sallis JF *et al*: **International physical activity questionnaire: 12-country reliability and validity**. *Med Sci Sports Exerc* 2003, **35**(8):1381-1395.
